# Adjuvant screening of the Senecavirus A inactivated vaccine in mice and evaluation of its immunogenicity in pigs

**DOI:** 10.1186/s12917-024-03949-5

**Published:** 2024-03-06

**Authors:** Jinyong Zhang, Peng Wang, Zhuoxin Li, Yubiao Xie, Ningyi Jin, He Zhang, Huijun Lu, Jicheng Han

**Affiliations:** 1 Academician Workstation of Jilin Province, Changchun University of Chinese Medicine, Changchun, China; 2 Changchun Veterinary Research Institute, Chinese Academy of Agricultural Sciences, Changchun, China

**Keywords:** Senecavirus A (SVA), Inactive vaccine, Adjuvants, Protection efficacy

## Abstract

**Background:**

Senecavirus A (SVA) causes an emerging vesicular disease (VD) with clinical symptoms indistinguishable from other vesicular diseases, including vesicular stomatitis (VS), foot-and-mouth disease (FMD), and swine vesicular disease (SVD). Currently, SVA outbreaks have been reported in Canada, the U.S.A, Brazil, Thailand, Vietnam, Colombia, and China. Based on the experience of prevention and control of FMDV, vaccines are the best means to prevent SVA transmission.

**Results:**

After preparing an SVA inactivated vaccine (CH-GX-01-2019), we evaluated the immunogenicity of the SVA inactivated vaccine mixed with Imject® Alum (SVA + AL) or Montanide ISA 201 (SVA + 201) adjuvant in mice, as well as the immunogenicity of the SVA inactivated vaccine combined with Montanide ISA 201 adjuvant in post-weaned pigs. The results of the mouse experiment showed that the immune effects in the SVA + 201 group were superior to that in the SVA + AL group. Results from pigs immunized with SVA inactivated vaccine combined with Montanide ISA 201 showed that the immune effects were largely consistent between the SVA-H group (200 µg) and SVA-L group (50 µg); the viral load in tissues and blood was significantly reduced and no clinical symptoms occurred in the vaccinated pigs.

**Conclusions:**

Montanide ISA 201 is a better adjuvant choice than the Imject® Alum adjuvant in the SVA inactivated vaccine preparation, and the CH-GX-01-2019 SVA inactivated vaccine can provide effective protection for pigs.

**Supplementary Information:**

The online version contains supplementary material available at 10.1186/s12917-024-03949-5.

## Background

Senecavirus A (SVA), also named Seneca Valley virus (SVV), is a non-enveloped, single-stranded, positive-sense RNA virus, which belongs to the genus *Senecavirus*, family of *Picornaviridae* (International Committee on Taxonomy of Viruses, http://www.ictvonline.com) [1, 2]. SVA was first reported as a contaminant from PER.C6 cells in 2002 [3]. A reported traceability to SVA suggests that it may have existed in U.S. pig populations for at least 30 years [4]; however, pigs infected with SVA were first reported in Canada in 2007 [5], and then cases of pigs infected with SVA were reported in Brazil [6], U.S. [7, 8], China [9], Colombia [10], Thailand [11], Vietnam [12], Mexico [4], and Chile [4]. In China, SVA cases were reported in Guangdong province in 2015 [9]; following this, several provinces, including Hubei, Henan, Heilongjiang, Fujian, and Shandong, successively reported cases of SVA-infected swine herds [13]. Several reports have shown that SVA can be detected and isolated in mice, mouse feces, and the environment of the infected, while, SVA nucleic acids can be detected in houseflies and *culicoides* [7, 13]. However, no previous study has provided evidence to prove the transmission route of SVA.

Typical clinical signs of pigs infected with SVA include vesicular and/or ulcerative lesions on the snout, oral mucosa, coronary bands, and hooves, which are indistinguishable from the clinical signs of pigs infected with vesicular stomatitis virus (VSV), foot-and-mouth disease virus (FMDV), and swine vesicular disease virus (SVDV) [14, 15]. As a cause of vesicular disease, both FMDV and SVA belong to the family *Picornaviridae*. FMDV is regulated by the World Organization for Animal Health (OIE) and is associated with economic loss (including production loss, trade restrictions, costs of regaining FMD disease-free area) [16]. Regarding control and prevention measure for FMDV, vaccines may be a measure to control the spread of SVA. As of now, no commercial vaccines for SVA are approved in China. Several vaccines for SVA have been reported in previous studies, including live attenuated vaccines, inactivated vaccines, and virus-like particle vaccines, with the results demonstrating that vaccinated animals are provided with complete protection [16–19].

In the current study, we prepared an SVA inactivated vaccine, and evaluated the immunogenicity of the SVA inactivated vaccine combined with Montanide ISA 201 and Imject® Alum adjuvants in mice. We also evaluated the immunogenicity of the SVA inactivated vaccine combined with ISA201 adjuvant in post-weaned pigs.

## Results

### Evaluation of the immunogenicity of the SVA inactivated vaccine in mice

**Fig. 1:**
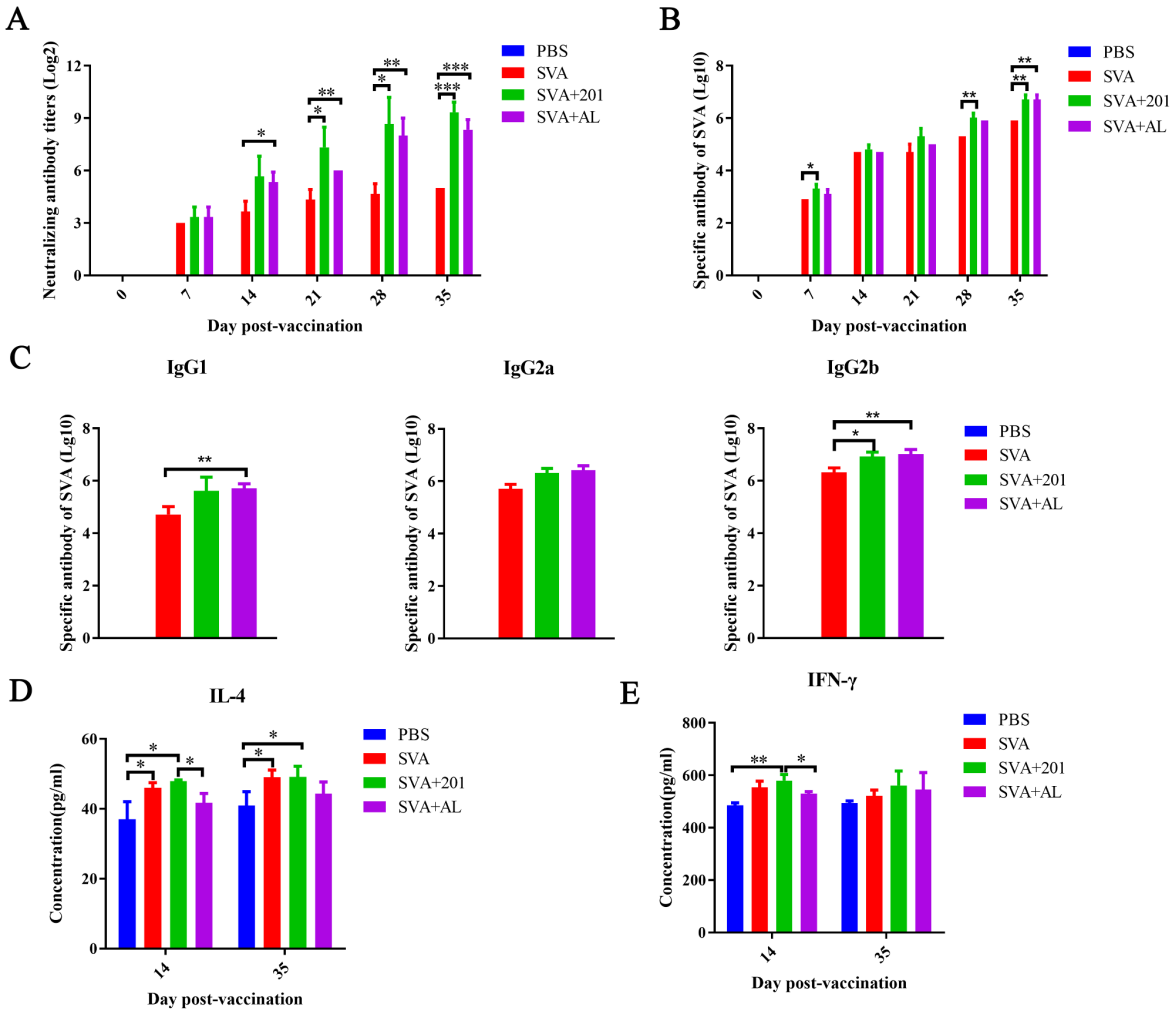
Evaluation of the immunogenicity of the SVA inactivated vaccine in mice. (**A**) Neutralizing antibody responses of mice to SVA vaccine vaccination. (**B**) Total IgG antibody responses of mice to SVA vaccination. (**C**) IgG subtype detection. IgG1 antibody (left), IgG2a antibody (middle), and IgG2b antibody (right) responses of mice to SVA vaccination. (**D**) Concentration of IL-4 detected in mice. (**E**) Concentration of IFN-γ detected in mice

Serum samples were collected at 0, 7, 14, 21, 28, and 35 days post-vaccination (dpv), and the titers of the neutralizing antibodies were detected. The results showed that in mice immunized with the SVA inactivated vaccine, the titers of the neutralizing antibodies showed an increasing trend, and the titers of the SVA mixed with ISA 201 group (SVA + 201) were higher than those in the SVA mixed with ISA Imject® Alum group (SVA + AL), although the difference was not significant. At 21, 28, and 35 dpv, the titers of neutralizing antibodies in the SVA + 201 and SVA + AL groups were significantly higher than those in the SVA group (Fig. 1A).

Serum samples were collected at 0, 7, 14, 21, 28, and 35 dpv, and the total IgG antibodies were detected. The results showed that the titers of total IgG antibodies were increased in the SVA + 201, SVA + AL and SVA groups; and prior 28 dpv, SVA + 201 group slightly higher than that in the SVA + AL group; however, at 35 dpv, the titers were similar in the SVA + 201 and SVA + AL groups (Fig. 1B).

Serum samples were collected at 35 dpv, and the titers of IgG1, IgG2a, and IgG2b were detected. The results showed that the titers of IgG1 in the SVA + 201 and SVA + AL groups almost were similar, but higher than those in the SVA group (Fig. 1C, left). The titers of IgG2a in the SVA + 201 and SVA + AL groups were similar, but higher than those in the SVA group (Fig. 1C, middle). The titers of IgG2b in the SVA + 201 and SVA + AL groups were similar, and significantly higher than those in the SVA group (Fig. 1C, right).

Serum samples were collected at 14 and 35 dpv, and the concentrations of interleukin 4 (IL-4) and interferon gamma (IFN-γ) were detected using the ELISA kit. The concentration of IL-4 in the SVA + 201 group was higher than that in the SVA + AL group at 14 and 35 dpv, and the concentration of IL-4 in the SVA group was generally consistent with that in the SVA + 201 group (Fig. 1D). The concentration of IFN-γ in the SVA + 201 group was higher than that in the SVA + AL group at 14 dpv (*P* < 0.01), while the concentration in the SVA + 201 group were generally consistent with that in the SVA + AL group at 35 dpv (Fig. 1E).

### Evaluation of the immunogenicity of the SVA inactivated vaccine in pigs

**Fig. 2:**
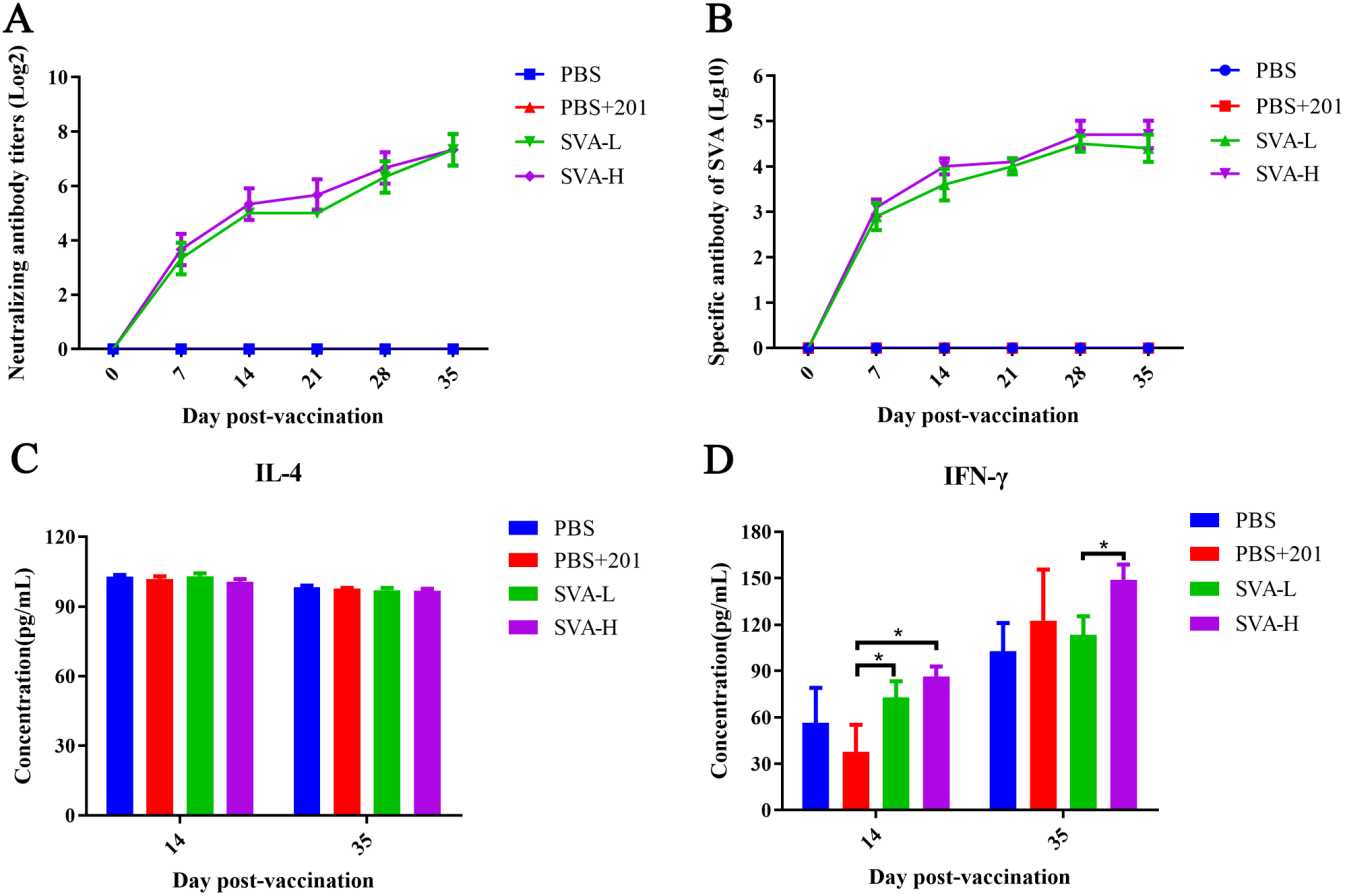
Evaluation of the immunogenicity of the SVA inactivated vaccine in pigs. (**A**) Neutralizing antibody responses of pigs to vaccination with the SVA vaccine. (**B**) Total IgG antibody responses of pigs to vaccination with the SVA vaccine. (**C**) Concentration of IL-4 detected in pigs. (**D**) Concentration of IFN-γ detected in pigs

Serum samples were collected at 0, 7, 14, 21, 28 and 35 dpv, and neutralizing antibodies were detected. The levels of neutralizing antibodies were slightly higher in the 200 µg antigen mixed with ISA 201group (SVA-H) than in the 50 µg antigen mixed with ISA 201group (SVA-L) at 7, 14, 21, and 28 dpv; however, the neutralizing antibodies reached the highest value and showed no significant difference in both the SVA-L and SVA-H groups at 35 dpv (Fig. 2A).

Serum samples were collected at 0, 7, 14, 21, 28, and 35 dpv, and total IgG antibodies were detected. The results showed that the total IgG antibodies could be detected as early as 7 dpv, while the titers peaked at 28–35 dpv. The total IgG antibody titers of the SVA-H group were slightly higher than those of the SVA-L group (Fig. 2B).

The serum samples were collected at 14 and 35 dpv, and the concentrations of IL-4 and IFN-γ were detected using an ELISA kit. The concentration of IL-4 showed no significant difference between groups (Fig. 2C). Moreover, the SVA-H group pigs were found to produce a higher concentration of IFN-γ than those in the others group (Fig. 2D).

### Protection against viral challenge in pigs

**Fig. 3:**
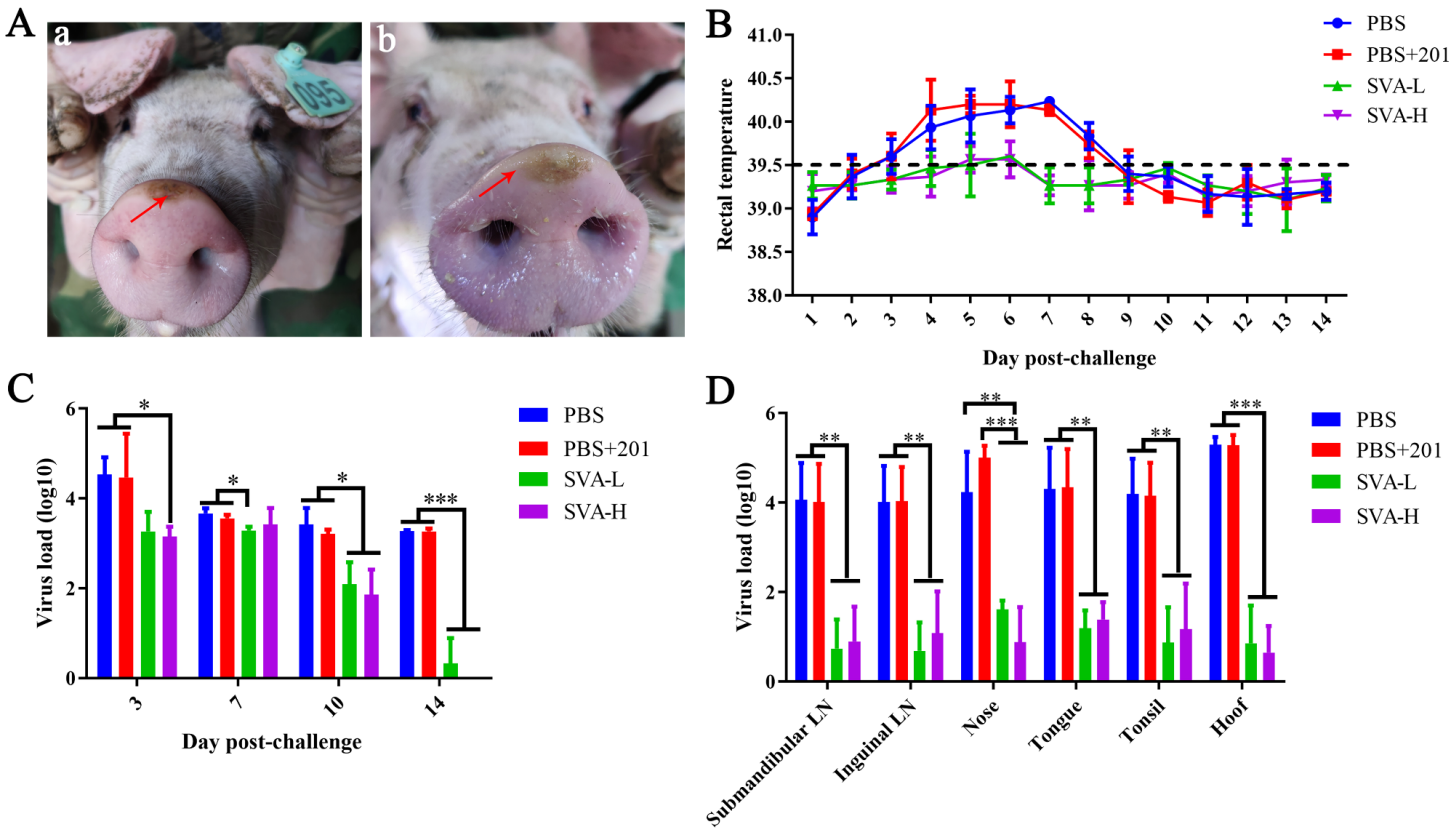
Protection against virus challenge in pigs. (**A**) Blisters on the noses of pigs in the PBS group (a) and PBS + 201 group (b) after being challenged with SVA. (**B**) The rectal temperature of each group of pigs following challenge with SVA. (**C**) Viremia detection. (**D**) Virus loads in the tissues

In pigs challenged with the CH-GX-01-2019 SVA strain as previously described [13], the clinical symptoms were carefully observed and recorded. Blisters were observed on the nose when pigs were challenged with SVA on day 4 post-challenge (dpc) in the phosphate buffered saline (PBS) group (Fig. 3A-a) and PBS + 201 group (Fig. 3A-b), while no distinct lesions were observed in the SVA-H and SVA-L groups, whether on the nose, hooves, or other tissues. The rectal temperature of the challenged pigs in the PBS and PBS + 201 groups was higher than 39.5℃ at 3–8 dpc, while that of the SVA-H and SVA-L group pigs was slightly elevated at 5–6 dpc (Fig. 3B).

The results of viremia detection showed that the virus load in the SVA-H and SVA-L groups was significantly reduced after the pigs were challenged with SVA on the 10 and 14 dpc compared to that observed in the PBS and PBS + 201 groups post-challenge. Furthermore, the virus could not be detected in the SVA-H group on the 14 dpc (Fig. 3C). The submaxillary lymph nodes (submaxillary LN), inguinal lymph nodes (inguinal LN), intestine, tongue, tonsil and hoof (with blister) were collected at 14 dpc. The virus loads of these tissues were significantly reduced in the SVA-H and SVA-L groups compared to those in the PBS and PBS + 201 groups (Fig. 3D).

## Discussion

Vaccines play a critical role in controlling and preventing various infectious diseases [20]. Inactivated vaccines have been applied in vaccine development for various epidemics, with advantages including not replicating in the host, mature manufacturing process and large quantities can be prepared in a short time. Due to these factors, inactivated vaccines are widely used for the prevention and control of various diseases, including severe acute respiratory syndrome coronavirus 2 (SARS-CoV-2) and FMD (the same virus family as SVA) [21, 22]. Pig infection with SVA was first reported in China in 2015, and data from epidemiological surveillance have shown that only four provinces have not yet reported SVA [23, 24]. Due to the continuous long-term spread and constant mutational reorganization, the SVA control method seems to be complicated and difficult [25]. As no methods have been approved to prevent and control SVA, it has caused economic losses to the pig farming industry in China. The SVA inactivated vaccine may represent an effective option to prevent and control the spread of SVA.

Formalin, binary ethylenimine (BEI), and β-Propiolactone (BPL) are often used for viral inactivation [16, 18, 26, 27]. Among these inactivators, BEI is widely used in FMD vaccine development [28] and was selected for inactivating the CH-GX-01-2019 SVA strain. Previous studies have shown that mice represent a good candidate animal model for SVA vaccine immunogenicity evaluation [26]. Adjuvants are important components in vaccine development, and effective adjuvant components can increase immune responses [29]. Adjuvants increase the immune response by localizing antigen for an extended time and attracting the appropriate cells (T cells, B cells, and antigen presenting cells) to interact with the immunogen and each other [30]. Here, Imject® Alum and ISA 201 adjuvants were used for the SVA inactivated vaccine in the mouse, and the neutralizing antibodies in the SVA IN + 201 group were found to be higher than those in the SVA IN + AL group. Helper T cells (Th cells) can differentiate into Th1 and Th2. IL-4 and IFN-γ induce the differentiation of Th0 cells into Th2 and Th1, which can regulate humoral and cellular immune responses [31–33]. Our results revealed that, compared to the SVA + AL group, higher levels of IL-4 and IFN-γ were produced in the SVA + 201 group.

In another study, the SVA CH-HNCY-2019 strain was prepared as an inactivated vaccine was emulsified with ISA 201 adjuvant. The results showed that mice (2-week-old female BALB/c) immunized with 10^7^ TCID_50_ produced titers of neutralizing antibodies were 1:32–1:64 and 1:64–1:128 at 14 and 28 dpv [26]. The neutralizing antibody titers (with titers 1:64–1:128 at 28 dpv) in our research were higher at 28 days than SVA CH-HNCY-2019 inactivated strain vaccine. The titer of neutralizing antibodies may be related to the quantity and purity of the SVA antigen, but our data also indicate that an immunity boost can increase the titer. These data confirm that the immunity of the CH-GX-01-2019 SVA inactivated vaccine mixed with ISA 201 is superior to that of the vaccine mixed with Imject® Alum adjuvants. In the pre-experiments in pigs, in which 100 µg SVA inactivated vaccine was mixed with ISA 201 or Imject® Alum adjuvants, the difference in neutralizing antibodies in the Imject® Alum adjuvant was very significant in the same group, but the value was stable in the ISA 201 adjuvant group (data shown in supplementary materials).

Yang et al. developed an SVA CH-FJ-2017 inactivated vaccine mixed with Montanide ISA 206 and proved that it could defend against challenge by SVA in finishing pigs [27]. Compared to the results of our study, this study adopted roller bottles to propagate the SVA so as to obtain a higher titer of SVA and high-purity virus particles; therefore, 2 µg SVA antigen can provide complete protection for the pigs. Li developed an SVA GD-ZYY02-2018 inactivated vaccine mixed and emulsified with adjuvant ISA 201, a high titer of anti-SVA neutralizing antibody (approximately 1:64 at 14 dpv, approximately 1:256 at 28 dpv) that can be detected in immunized pigs (immunized with 3 mL/10^8.25^ TCID_50_/mL), which was found to protect finishing pigs against the challenge of homologous virus [34]. Liu et al. inactivated the LNSY01-2017 SVA strain, their results showed that β-propiolactone (BPL) may be a better inactivator, and also in the results of the adjuvants indicating MONTANIDETM IMG 1313 can provide a better protection efficacy than ISA 201 [35]. Furthermore, Buckley et al. developed an SVA inactivated vaccine mixed with an oil-in-water adjuvant and a whole-virus inactivated SVA vaccine against challenge in nursery-aged pigs and mature sows to assess the protection of passive maternal immunity generated by immunized dams [16]. These studies offer promising inactivated vaccine candidates; however, the immune effects are affected by different virus strains, preparation processes, adjuvants, immune doses, immunity boost and other factors. In our study, the ISA 201 adjuvant was superior to the Imject® Alum adjuvant in the mouse experiment. In the pig experiment, the SVA inactivated vaccine (mixed with ISA 201 adjuvant) can stimulate pigs to produce high levels of neutralizing and total IgG antibodies and stimulate the secretion of IFN-γ. And the pigs immunized with inactivated SVA vaccine exhibited significantly reduced viremia and the virus load in tissues, while protecting them from clinical symptoms. These findings provide data for researchers to develop vaccines to prevent the spread of SVA.

## Conclusion

In this research, the immune effect results showed that mice immunized with SVA inactivated vaccine mixed with ISA 201 adjuvants were superior to those immunized with Imject® Alum adjuvants. Good immune effects were produced when pigs immunized with inactivated vaccine mixed with ISA 201 adjuvants, with the vaccinated groups demonstrated significantly reduced viremia and viral load in tissues, protecting pigs from the clinical symptoms resulting from SVA challenge. As an inactivated vaccine, the CH-GX-01-2019 strain provides complete protection to pigs.

## Materials and methods

### Virus propagation and inactivation

The SVA strain CH-GX-01-2019 was previously isolated from Guangxi Province, China, by our laboratory group [36]. Baby hamster kidney cells (BHK-21) were maintained in Dulbecco’s modified Eagle’s medium (DMEM; Hyclone, Logan, UT, USA) supplemented with 5% fetal bovine serum (FBS; Hyclone, Logan, UT, USA) and cultured at 37℃ under 5% CO_2_ to propagate and titer SVA.

SVA was mass cultured, the cell debris was removed by centrifugation, and the supernatant was retained for inactivation. A 2% volume of 0.1 mol BEI was added to SVA for 12 h at 30 ℃ with constant shaking, and then a 2% volume of 0.1 mol BEI was added again for 14 h at 30 ℃ with constant shaking. The inactivation reaction was stopped by adding 1 M sodium thiosulfate. Inactivated SVA was inoculated into BHK-21 cells, and the cytopathic effect (CPE) and indirect immunofluorescence assay (IFA) were used to prove the effect of the inactive virus.

### Formulation of the inactivated vaccine

The inactive virus was purified using a sucrose density gradient (15–65%) after centrifugation at 30,000 rpm for 120 min at 4℃. The quantity of inactivated virus was measured using an Enhanced BCA Protein Assay Kit (Beyotime, Shanghai, China) according to the manufacturer’s instructions. Montanide ISA 201 (SEPPIC, Paris, France) and Imject® Alum (Thermo Fisher, Massachusetts, USA) were selected as the adjuvants to evaluate the immune effects in mice, and Montanide ISA 201 was selected as the adjuvant to evaluate the immune effects in pigs. The volume ratio of the inactive virus to the ISA 201 adjuvant was 4.5:5.5, while the volume ratio of the inactive virus to the Imject® Alum adjuvant was 3:1.

### Animals and immunization experiments

**Fig. 4:**
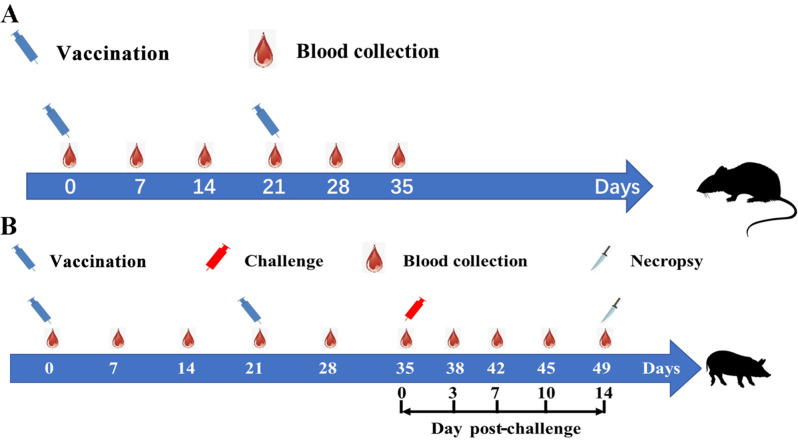
Experimental design of animal immunization with SVA inactivated vaccine. (**A**) Experimental design of mouse immunization with SVA inactivated vaccine. (**B**) Experimental design of pig immunization with SVA inactivated vaccine

Thirty-two mice (C57BL/6, 5-week-old, female) (purchased from Jilin GENET-MED Biotechnology Co., Ltd., Changchun, China) were randomly divided into four groups with eight mice in each group: PBS control group (PBS); SVA inactivated vaccine group (SVA), SVA inactivated vaccine with Imject® Alum (SVA + AL), and SVA inactivated vaccine with Montanide ISA 201 (SVA + 201). The mice were immunized with 50 µg antigen (200 µL) immunized intramuscularly in the leg at day 0, and the immunization was boosted on day 21 (Fig. 4A).

Twenty post-weaned pigs (5 weeks old) were purchased from a farm in Changchun, China. The nucleic acids of ASFV, PRRSV, PEDV, FMDV, SVA, VSV, VESV, SVDV, and CSFV were negative in these pigs, and all of the pigs were seronegative for SVA. These 20 pigs were randomly divided into four groups, with each group containing five pigs: PBS control group (PBS), 50 µg antigen mixed with ISA 201group (SVA-L), and 200 µg antigen mixed with ISA 201group (SVA-H). The pigs were immunized with antigen (2 mL) intramuscularly in the neck on day 0, and the immunization was boosted on day 21 (Fig. 4B).

### Neutralization assay

Serum samples were collected at 0, 7, 14, 21, 28, and 35 days-post-immunization (dpv). Neutralizing antibody titers in the serum of mice or pigs immunized with vaccine were detected by the virus neutralizing antibody test (VNT). The VNT was performed as described previously [13, 27]. The serum samples were inactivated at 56℃ for 30 min, with two-fold dilutions of inactive serum with DMEM added into 96-well plates. Next, 50 µL of diluted serum samples were incubated with 50 µL 200 TCID_50_ CH-GX-01-2019 at 37 °C for 1 h, before adding 100 µL BHK-21 cells (1 × 10^5^) were added into each well of the 96-well plates and further incubating at 37 °C for 72 h. Neutralizing antibody titers were determined via observation of the CPE in BHK-21 cells. The neutralizing antibody titers against the SVA CH-GX-01-2019 strain were calculated and expressed as the log2 of the reciprocal of the highest serum dilution that inhibits 100% of SVA infection/replication in the culture wells [35].

### Specific antibody assay

#### Total IgG-specific antibody assay

Serum samples were collected at 0, 7, 14, 21, 28, and 35 dpv. Total IgG was detected by enzyme-linked immunosorbent assay (ELISA). The inactivated viruses were coated with coating buffer (Solarbio Life Science, Beijing, China) and incubated overnight at 4℃. The coated wells were blocked with 5% skim milk and incubated at 37℃ for 1 h, discarding the skim milk, TBST was used to wash the coated wells twice. The serum samples (experimental groups and negative controls) were diluted with PBS (Solarbio Life Science, Beijing, China), added into the wells of ELISA plates, and incubated at 37℃ for 1 h. The serum samples were removed from the coated wells, and then TBST was used to wash the wells (three times, shaking for 3 min). HRP goat anti-mouse antibody (Beyotime Biotechnology, Shanghai, China) was used as the secondary antibody (1:10000) and incubated at 37℃ for 30 min. The wells were washed with TBST five times (shaking for 3 min). TMB solution (Solarbio Life Science, Beijing, China) was added into the wells, incubated at room temperature for 15 min (keep in the dark), and then the reaction was stopped by stop buffer (Solarbio life science, Beijing, China). The absorbance was read at 450 nm (OD value). The titers of the antibody were calculated when S/*P* ≥ 2.1 (S/P = OD_450 nm_ of experimental groups / OD_450 nm_ of negative controls).

#### IgG subtype-specific antibody assay

Serum samples were collected at 35 dpv. The performance of the IgG subtype-specific antibody assay was as described in section of total IgG-specific antibody assay, but with HRP-conjugated goat anti-mouse IgG1, IgG2a, or IgG2b used as the secondary antibody (1:20000).

### Cytokine detection

Serum samples were collected at 14 and 35 dpv. IL-4 and IFN-γ were detected using the ELISA kit, performed according to the manufacturer’s instructions. Cytokine test kits for IL-4 and IFN-γ(mouse derived) were purchased from Invitrogen (Shanghai, China), and cytokine test kits for IL-4 and IFN-γ (pig derived) were purchased from Cloud-clone (Wuhan, China).

### Protection against viral challenge in pigs

At 35 dpv in pigs, the pigs were challenged with 10 mL SVA (CH-GX-01-2019, virus titer of 10^7.5^TCID50/mL), via administration of 5 mL into each nostril [13]. The clinical symptoms and rectal temperatures were monitored for 14 days. Blood samples were collected at 3, 7, 10, and 14 days post-challenge (dpc) for virus load detection by SYBR Green I quantitative real-time PCR, as previously described [13]. The submaxillary lymph nodes (submaxillary LN), inguinal lymph nodes (inguinal LN), intestine, tongue, tonsil and hoof (with blister) were collected at 14 dpc, and virus loads were detected as previously described [13].

### Statistics

Data were analyzed using GraphPad Prism (version 6.0) software (GraphPad Software Inc., La Jolla, CA). All data are presented as the mean ± SD. Differences in levels between the different groups were determined by one-way repeated measures ANOVA and least significant difference (LSD). Differences were considered statistically significant when *P* < 0.05 (* indicates *P* < 0.05), *P* < 0.01 (** indicates *P* < 0.01) and *P* < 0.0001 (** indicates *P* < 0.0001).

### Electronic supplementary material

Below is the link to the electronic supplementary material.

**Additional file**: Neutralizing antibodies analysis in the pre-experiments in pigs. **Table S1** Neutralizing antibody titers of pigs immunized with SVA inactivated vaccine. **Figure S1** Neutralizing antibody titers of pigs immunized with SVA inactivated vaccine.

## Data Availability

All data analyzed during this study are included in this published article. The raw data generated during the study are available from the corresponding author on reasonable request.

## Abbreviations

SVA: Senecavirus A
SVV: Seneca Valley virus
VSV: Vesicular stomatitis virus
FMDV: Foot-and-mouth disease virus
SVDV: Swine vesicular disease virus
BEI: Binary ethylenimine
IFA: Indirect immunofluorescence assay
dpv: Day post-vaccination
dpc: Day post-challenge
