# Supplementary material for: Adjuvant screening of the Senecavirus A inactivated vaccine in mice and evaluation of its immunogenicity in pigs
Source: BMC Vet Res. 2024 Mar 6;20:82. doi: 10.1186/s12917-024-03949-5 (PMC10916230; doi:10.1186/s12917-024-03949-5)
Supplement: Supplementary file 1 — Additional file: Neutralizing antibodies analysis in the pre-experiments in pigs. Table S1 Neutralizing antibody titers of pigs immunized with SVA inactivated vaccine. Figure S1 Neutralizing antibody titers of pigs immunized with SVA inactivated vaccine. [file 12917_2024_3949_MOESM1_ESM.docx]

Neutralizing antibodies analysis in the pre-experiments in pigs

Table S1 Neutralizing antibody titers of pigs immunized with SVA inactivated vaccine

| Groups | Pig No. | 0dpi | 7dpi | 14dpi | 21dpi | 28dpi |
| --- | --- | --- | --- | --- | --- | --- |
| PBS | 1 | 0 | 0 | 0 | 0 | 0 |
|  | 2 | 0 | 0 | 0 | 0 | 0 |
|  | 3 | 0 | 0 | 0 | 0 | 0 |
|  | 4 | 0 | 0 | 0 | 0 | 0 |
|  | 5 | 0 | 0 | 0 | 0 | 0 |
| SVA-IN-Al-Pigs | 6 | 0 | 1:32 | 1:64 | 1:32 | 1:512 |
|  | 7 | 0 | 1:2 | 1:8 | 1:8 | 1:8 |
|  | 8 | 0 | 1:16 | 1:16 | 1:32 | 1:256 |
|  | 9 | 0 | 1:8 | 1:16 | 1:16 | 1:64 |
|  | 10 | 0 | 1:8 | 1:8 | 1:64 | 1:128 |
| SVA-IN-201-Pigs | 11 | 0 | 1:16 | 1:64 | 1:128 | 1:256 |
|  | 12 | 0 | 1:16 | 1:32 | 1:64 | 1:128 |
|  | 13 | 0 | 1:16 | 1:32 | 1:64 | 1:128 |
|  | 14 | 0 | 1:16 | 1:64 | 1:64 | 1:128 |
|  | 15 | 0 | 1:32 | 1:64 | 1:128 | 1:128 |

Note: dpi means days-post-immunization; SVA-IN-Al-Pigs means pigs immunized with SVA inactivated vaccine combined with Imject® Alum adjuvant; SVA-IN-201-Pigs means pigs immunized with SVA inactivated vaccine combined with Montanide ISA 201.


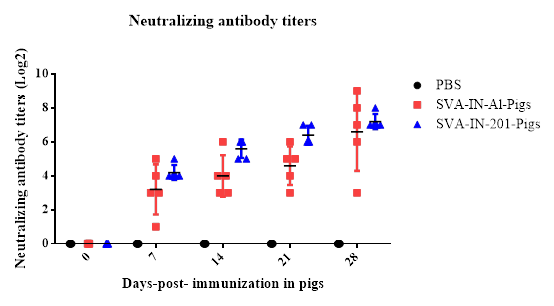


Figure S1 Neutralizing antibody titers of pigs immunized with SVA inactivated vaccine
